# Curating a Case Catalog: Development and Implementation of a Process for Revising Small Group Teaching Cases for Pre-clerkship Medical Education

**DOI:** 10.1007/s40670-022-01681-z

**Published:** 2022-12-07

**Authors:** Lia Pierson Bruner, Brett Szymik, Ellen House, M. Tresa Chappell, Dina Teshager, Amy Baldwin

**Affiliations:** 1 Department of Family and Community Medicine, Augusta University/University of Georgia Medical Partnership, UGA Health Sciences Campus, Russell Hall, Room 235K, 1425 Prince Avenue, Athens, GA 30602 USA; 2 Department of Cellular Biology and Anatomy, Augusta University/University of Georgia Medical Partnership, Athens, GA USA; 3 Department of Psychiatry, Augusta University/University of Georgia Medical Partnership, Athens, GA USA; 4 Department of Pediatrics, Augusta University/University of Georgia Medical Partnership, Athens, GA USA; 5 Augusta University/University of Georgia Medical Partnership, Athens, GA USA; 6 Department of Biochemistry and Molecular Biology, Augusta University/University of Georgia Medical Partnership, Athens, GA USA; 7 School of Social Work, University of Georgia, Athens, GA USA

**Keywords:** Case-based learning, Undergraduate medical education, Case revision, Diversity and inclusion, Patient-centered

## Abstract

Small group, case-based learning (CBL) is an integral component of many pre-clerkship undergraduate medical education (UME) curricula. We report here an institutional process for curating a catalog of CBL cases utilized in a pre-clerkship curriculum, providing a practical guide for faculty. We describe the structured revision process conducted by a team of foundational and clinical science faculty, which incorporates student and faculty feedback. Revisions take into account core attributes of a case catalog, producing a collection of cases that are more relevant and instructional, realistic, challenging, consistent, current, diverse and inclusive, patient-centered, and mission-centered. Measurable outcomes after implementation of this process include increased focus on primary care as well as humanization and diversification of the case patients.

## Introduction

Case-based learning (CBL) is widely utilized in healthcare education around the world, and students use inquiry-based learning to prepare for clinical practice by applying knowledge to clinical cases with the goal of linking theory to practice [1, 2]. Individual institutions often encounter the challenge of developing CBL cases to meet their curricular needs. There are published resources in the education literature that are helpful in developing and reviewing effective individual CBL cases [3, 4]. There are also a number of published resources on addressing bias, diversity, inclusion, and equity in medical education [5–12]. However, there do not appear to be any available, published resources that help guide faculty in curating a catalog of CBL cases over the course of students’ medical training.

One review of 100 studies of case-based teaching across disciplines revealed five core attributes to provide a framework for educators to develop effective cases for use in higher education: relevant, realistic, engaging, challenging, and instructional [3]. While many of the attributes of individual cases can be applied to case catalogs, faculty at our medical school felt it was important to include additional attributes that reflect many efforts in medical education to provide more holistic, patient-centered, diverse, and inclusive curricula.

Prior to the development of an institutional process, most of the cases utilized in our pre-clerkship curriculum were over 10 years old, and many were based on the personal experiences of an internal medicine physician, skewing our case catalog towards hospitalized adults. We lacked a process for making needed changes to our CBL cases to optimize the case catalog to better meet the goals of our institution. In addition, we had no effective mechanism to collect or incorporate faculty or student feedback on these cases. To address these issues, we developed and describe here a collaborative process for revising cases and tracking the contents of a catalog of pre-clerkship cases, including establishment and utilization of case catalog core attributes. This process allows cases to evolve with the larger curriculum to match the ever-changing world of science and medicine.

## Approach

The Augusta University/University of Georgia Medical Partnership, a 4-year campus of the Medical College of Georgia, opened in 2010. We are a teaching-focused campus of Georgia’s only public medical school, and we have foundational and clinical science faculty working together in an increasingly integrated curriculum. For the first decade, 40 students entered our campus each year, and now our class size has increased to 60. CBL is the core component of the integrated, systems-based, modular pre-clerkship curriculum, where students learn from cases throughout their first and second years in three 2-h sessions each week. Students work through one case per week in year 1 and two cases per week in year 2. Each CBL group of seven to eight students is facilitated by a pair of clinical and foundational science educators.

Most cases in our catalog are utilized each year. The cases are designed to progress over the course of each week, and correspond to the curricular theme of the week. Given the central nature of CBL cases in our curriculum and the importance of case revisions, we needed an institutional process for curating a case catalog. We expect that our approach to continuously and intentionally update and oversee the maintenance of our catalog of cases will be widely applicable, as many medical schools utilize CBL in their curricula.

### Formation of a Case Oversight Team

A Case Oversight Team (COT) was created at the Medical Partnership in 2018. The Campus Associate Dean of Curriculum and Campus Dean charged the team with creating a process for revision, replacement, and maintenance of cases in our catalog. The COT is led by a chair and is composed of about ten clinical and foundational science faculty members with diverse experience and expertise. COT members are involved in all aspects of the curriculum, including lectures, team-based learning, flipped classroom sessions, anatomy lab, simulation, CBL, clinical skills, and service learning. COT members take turns being lead editors for groups of cases by topic or systems-based module. The diverse team ensures integration of our CBL component within the context of the overall curriculum.

The bulk of the case revisions are done by the lead editors in conjunction with the COT chair, drawing on faculty and student feedback as well as solicited input from content specialists and organizers of the individual systems-based modules. When possible, lead editors oversee similar content areas in both year 1 and year 2, to help with vertical integration and to avoid unwanted overlap in case details. The COT chair coordinates the process and helps maintain consistency across cases by participating in group editing and reviewing all revised cases prior to use.

### Student Feedback

Prior to the formation of the COT, there was no clear mechanism for student feedback on the cases. Students now have the opportunity to give group feedback each week by answering the following questions: “Which aspects of the case most effectively facilitated your learning, and why?” and “Which aspects of the case could be improved?” Answers are collected via Google Forms, collated, reviewed by the lead editors, shared with faculty, and used for future case modifications.

### Faculty Input

To provide an open and transparent way for pre-clerkship faculty members to contribute, an online Google platform is utilized to house the most recent versions of the cases. Faculty members have access to these cases and are encouraged to place comments and suggestions in the cases at any time. For each of our modules, an editing team is created annually, consisting of the COT lead editor(s) and COT chair as well as the faculty who organize the content for a given systems-based module.

### Case Revision and Writing

**Fig. 1:**
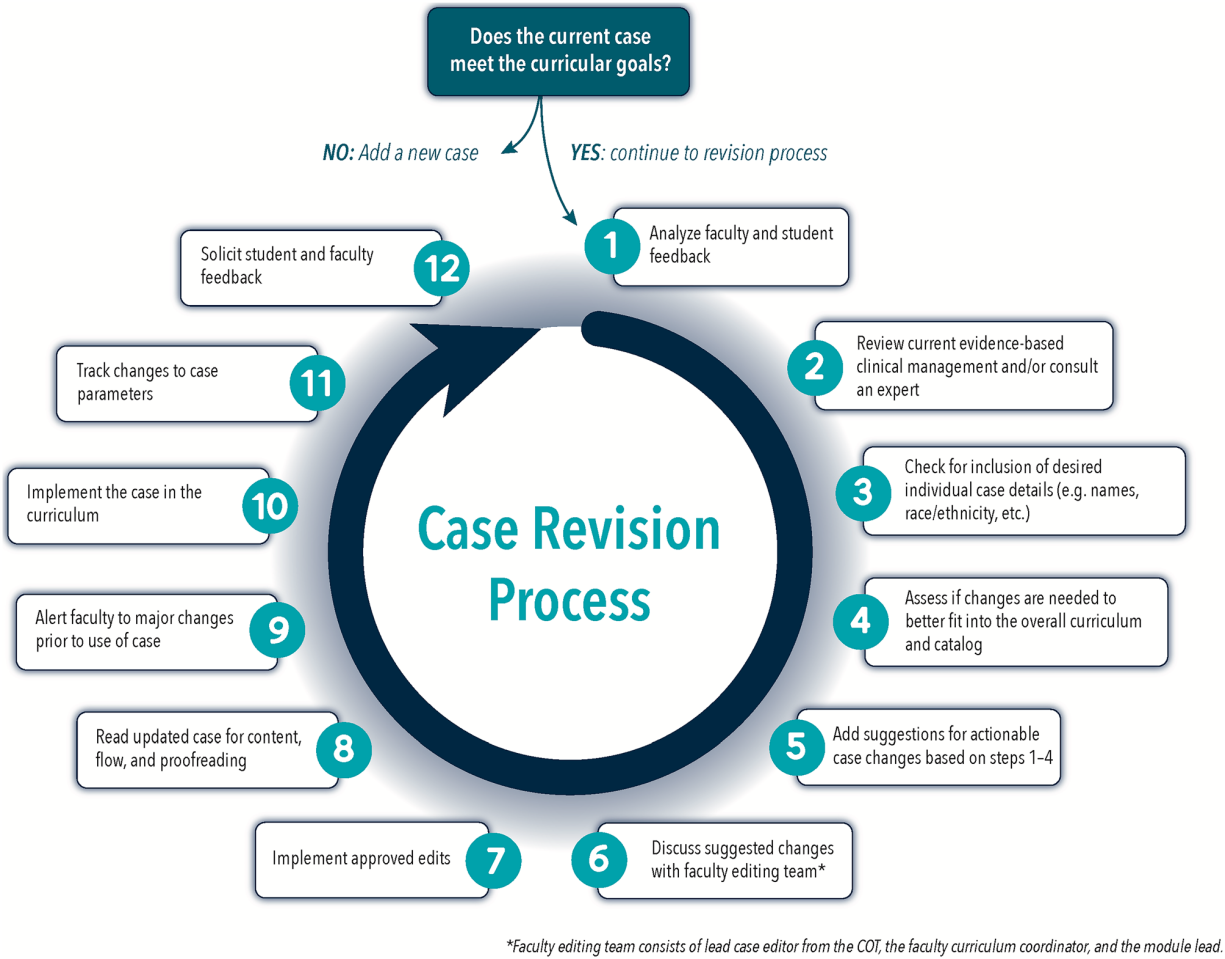
Process for revising small group teaching cases for pre-clerkship medical education

When evaluating a particular case, the first step is to decide if it currently meets curricular goals. For those cases that meet curricular goals, but may still need updating, a process for revision was developed iteratively based on a continuous quality improvement model (Fig. 1). In the first year of the COT, the main priorities were to update clinical management to the current standard of care (step 2) and to revise the physical exams to match the clinical skills curriculum. We also began soliciting feedback from faculty and students, which was utilized in the revisions for the following year, making these standard steps in the process (step 12 and step 1). During this time, our pre-clerkship curriculum for foundational sciences and clinical medicine were integrated, so the importance of assessing fit of the case (step 4) with the overall curriculum became even more important. To make sure that planned major changes to the cases are in line with each aspect of the curriculum, faculty editing meetings with the leaders of each module were added (step 6). As we began tracking case details (step 11), we targeted some systematic changes, necessitating a step to check for desired individual case details (step 3) and to assess if changes were needed to better fit the catalog (step 4). With many changes being made to the cases each year, it became clear that assessing for content and flow was also important (step 8). We ensured communication of case changes by sending emails to the faculty before the start of each module, alerting them to major changes before cases were implemented in CBL sessions (step 9).

When cases need to be replaced, faculty are encouraged to write new cases with COT members to meet curricular needs. The COT is ultimately responsible for the writing of any new cases. All COT members review each new case and vote on approval before a new case is implemented. A practical, internal guide for writing cases was also created, tailored to the case formats utilized in our curriculum, which differ between first and second year.

### Case Catalog

When the COT started, there was no convenient way to see summaries of the existing cases. A case catalog spreadsheet was created that clearly and comprehensively details specifics about each case including the patient’s chief concern, initial setting, student role, final diagnosis, as well as patient age, gender, sexual orientation, and race/ethnicity. This system documented a comprehensive and easy-to-reference summary of our entire case catalog over the pre-clerkship years, allowing more explicit links to both the module and weekly learning objectives, which has been quite helpful in the ongoing process of mapping our curriculum.

### Case Catalog Attributes

Case-based teaching is accepted as an effective instructional method to promote a learner's critical thinking skills, and five core attributes of individual cases have been synthesized to provide a framework for educators to develop effective cases for use in higher education: relevant, realistic, engaging, challenging, and instructional [3]. While almost all of these attributes for individual cases apply well to an overall case catalog, there are additional attributes that are critical for holistic student learning. Based on experience facilitating CBL cases, priorities in systematic editing, and pedagogical goals of our institution, we felt that the following additional attributes related to the overall case catalog were also needed: consistent, current, diverse and inclusive, patient-centered, and mission-centered. Some of these attributes, like diversity and inclusion, can only be partially addressed in any one teaching case and should be considered in the development and maintenance of a well-designed case catalog.

The attributes of being consistent and current were the main impetus for the creation of the COT. The COT was charged with helping to ensure that cases were *current* in terms of terminology, diagnostics, and treatment. Similarly, the COT was to make sure that the physical exam, communication skills, and pathophysiology components of the cases were *consistent* both across the catalog of cases as well as teaching elsewhere in the curriculum.

Being *diverse and inclusive* is another important goal, and one that can only be realized in the context of a collection of cases utilized over time in a curriculum. The Association of American Medical Colleges (AAMC) and the American Medical Association (AMA) have emphasized the importance of combating structural racism within medical education, and we wanted to ensure attention was paid to this and other equity issues [10–12]. *Patient-centered* care is ultimately the goal of medical education, so our catalog of cases needs to model holistic, empathetic care in a variety of scenarios that support discussions of ethics, social determinants of health, and the art of medicine.

Teaching cases in a catalog can be adjusted to support and reflect institution-specific missions, thus *mission-centered* is another core attribute. Each institution has its own mission and goals. Our institution was created with a mission to produce more primary care doctors for Georgia. Another institutional goal was to teach lifelong learning skills in order to meet the challenges of the twenty-first century practice of medicine. Establishing the COT has enabled us to better represent the mission and institutional goals of the Medical Partnership, adding an essential level of accountability, which is crucial as we are a unit of the only public medical school in Georgia.

Combining all of these attributes in a pre-clerkship case catalog ensures that an essential component of the curriculum is relevant and instructional, realistic, challenging, consistent, current, diverse and inclusive, patient-centered, and mission-centered. Implementation of the COT has allowed for the intentional creation, revision, and continued monitoring of this main curricular component. In summary, a case catalog should have the following attributes:

#### Relevant and Instructional

Contain a range of common and clinically important scenarios to support the curricular learning objectives

#### Realistic

Reflect the ever-changing landscape of medicine that students will encounter in clinical practice

#### Challenging

Increase complexity and pace over time to match and promote students’ intellectual development

#### Consistent

Reinforce both the foundational science and clinical skills taught in other portions of the curriculum

#### Current

Demonstrate up-to-date understanding of disease processes and evidence-based clinical management

#### Diverse and Inclusive

Reflect the diversity of patient populations and actively work to combat stereotypes and bias within healthcare

#### Patient-centered

Model holistic, empathetic care with scenarios that support varied discussions including professionalism, ethics, social determinants of health, and the art of medicine

#### Mission-centered

Support the goals of the institution

## Outcomes

The work of writing and revising cases now involves many faculty members rather than one or a small number of faculty. This empowers faculty and creates a sense of collective investment, utilizes the faculty’s diverse backgrounds, allows for timely and robust case updating, and decreases likelihood of inherent or unconscious biases being reflected in the cases. Having a system to track changes in the catalog over time has allowed us to be intentional with systematic changes and to target cases for heavy revision or replacement. It has also allowed us to identify missing elements as well as overlap amongst the cases. Diversification of case patient demographics is a priority of the revision process with a goal to combat implicit biases within medical education. We have seen and documented improvements in the case catalog core attributes after 3 years of COT editing.

### Relevant and Instructional

Since the creation of the COT, we have replaced 29 of the 111 cases (26.1%) in our catalog to avoid overlap and focus on common and clinically important scenarios to better achieve our curricular goals. The case revision process also focuses heavily on enriching the patient-doctor experience to be more relevant. Cases were updated with explicit settings and describe the student role as “you” to bring them in as active participants and allow greater role-play experiences for practicing communication skills such as shared decision-making, motivational interviewing, and delivering bad news.

### Realistic

By creating mechanisms for encouraging and incorporating faculty and student feedback, the COT has improved the realism of our cases. Tests and treatments have been modernized. More images, as well as video and audio demonstrations, have been included. The flow of the cases is more realistic, including handoffs and specialist consults. The COT also worked to boost the ethical and emotional complexity of the cases to better reflect the realities of clinical practice.

### Challenging

The revision process allows for progressive building of knowledge and skills with increasing complexity of the cases in the catalog throughout the pre-clerkship curriculum. Discussion prompts, included in the first semester in year 1, are removed as students are expected to become increasingly independent in their learning. Advanced topics (e.g., treatment decisions) are incorporated more frequently as students progress through the curriculum.

### Consistent

The COT has expanded efforts to make the cases consistent with other parts of the curriculum, including standardizing the physical examination methods and documentation to match those taught in clinical skills. All cases include facilitator notes that encourage faculty facilitators of the CBL group to ask questions about important concepts from the weekly learning objectives in a holistic, patient-centered manner. The editing process includes expansion and revision of these facilitator notes, helping to ensure that students have similar learning experiences despite differences between student teams and facilitators. Efforts have also been made to make the cases consistent across the catalog in terms of format, timing, and flow of information.

### Current

Cases have been heavily revised to be consistent with the current understanding of disease processes and evidence-based clinical management. All cases now receive at least minor updates annually, driven mostly by medical advancements, guideline updates, faculty and student feedback, and curricular changes.

### Diverse and Inclusive

The COT established the goal of having our case patient population be at least as diverse as the population of Georgia. In academic year (AY) 2016, only 11 cases (10.2%) explicitly identified the patient’s race. There was concern that students and faculty might inadvertently apply a racial background to the patients where it was not specified, potentially affecting learning issues and/or reinforcing biases [7]. By AY 2020, 106 cases (95.5%) explicitly stated the patient’s self-identified race in the social history, and the racial diversity of the case patients approached that of Georgia’s population [13]. We also introduced cases with transgender patients, increased the number of pediatric patients, and diversified sexual orientation (Table 1).

Additionally, we performed a careful review of the language used in the cases to ensure avoidance of historical and harmful stereotypes within healthcare. We moved mentions of race/ethnicity to the social history where these are now self-identified by the patient. We have also inserted more specific ancestry in some cases and removed the term Caucasian. We adjusted the case catalog so students and faculty encounter a diverse range of family structures, socioeconomic status, cultural and religious views, and patient abilities while being intentional to avoid perpetuating stereotypes. For example, the case of HIV infection now occurs in a cis-gender heterosexual man and our case of sickle cell disease occurs in a patient of Italian descent (sickle beta zero thalassemia), rather than in a patient of African descent, to highlight race-based diagnostic bias. We changed GFR calculations to the race-free calculation endorsed by the National Kidney Foundation, with discussion in the facilitator notes about risks of using race-based calculations [14]. We have included images consistent with the self-identified race of the patients whenever possible. Also, mental health, substance use disorders, and other potentially stigmatized conditions occur largely in non-minoritized patients and are routinely part of patient histories when presenting for other concerns.

### Patient-centered

All patients are now more multidimensional, and cases include expanded descriptions of their lives, greater inclusion of social determinants of health, and direct patient quotes. To humanize our patients, we sought to give them first and last names. In AY 2016, only five patients (4.6%) had both a first and last name; most year 1 patients were given only a first name and last initial, and year 2 patients had no names. By AY 2020, 110 patients (99.1%) were fully named (Table 1). Patient-doctor interactions were enriched with embedded demonstrations of communication skills, ethical complexity, and patient emotional experiences.

### Mission-centered

Our institution was founded with the express purpose of ameliorating the shortage of primary care doctors in Georgia. In AY 2016, only one case (0.9%) explicitly occurred in a primary care setting; 73 cases (67.6%) did not state the setting, but generally involved testing and treatments not readily available in the outpatient setting. By AY 2020, 99 cases (89.2%) clearly stated where the patient initially presented, and 46 cases (41.4%) began, and many ended, in primary care settings (Table 1). Cases now better emphasize the capabilities of primary care physicians while still exposing students to a greater variety of clinical settings and physician roles.

**Table 1:** Comparison of case characteristics from before (academic year 2016–2017, “AY 2016”) and after (academic year 2020–2021, “AY 2020”) institution of a Case Oversight Team

	**AY 2016**	**AY 2020**
	***n* (%)**	***n* (%)**
**Number of cases**^a^	108	111
**Patient has full name**		
Yes	5 (4.6)	110 (99.1)
No	103 (95.4)	1 (0.9)
**Age of patient**		
Pediatric (< 1–18 yrs)	8 (7.4)	14 (12.6)
Infant or child (< 1–5 yrs)	4 (3.7)	4 (3.6)
Older child (6–12 yrs)	2 (1.9)	7 (6.3)
Adolescent (13–18 yrs)	2 (1.9)	3 (2.7)
Adult (≥ 19 yrs)	100 (92.6)	97 (87.4)
Adult (19–65 yrs)	71 (65.7)	74 (66.7)
Elder (> 65 yrs)	29 (26.9)	23 (20.7)
**Gender of patient**		
Man	51 (47.2)	46 (41.4)
Woman	49 (45.4)	49 (44.1)
Boy	6 (5.6)	5 (4.5)
Girl	2 (1.9)	9 (8.1)
Transman	0	1 (0.9)
Transwoman	0	1 (0.9)
**Race of patient**		
Unstated	97 (89.8)	5 (4.5)
Black or African-American	6 (5.6)	25 (22.5)
East Asian	0	7 (6.3)
Multiracial	0	10 (9.0)
Native American or Alaska Native	0	2 (1.8)
South Asian	1 (0.9)	4 (3.6)
White	4 (3.7)	58 (52.3)
**Sexual orientation of patient**		
Unstated	44 (40.7)	36 (32.4)
Heterosexual	60 (55.6)	66 (59.5)
Homosexual	3 (2.8)	7 (6.3)
Bisexual	1 (0.9)	2 (1.8)
**Initial clinical setting of case**		
Unstated	73 (67.6)	12 (10.8)
Primary care	1 (0.9)	49 (44.1)
Family medicine clinic	0	21 (18.9)
General internal med. clinic	1 (0.9)	18 (16.2)
Pediatric clinic	0	7 (6.3)
Other^b^	0	3 (2.7)
Emergency department	12 (11.1)	24 (21.6)
Hospital general ward (inpatient)	22 (20.4)	10 (9.0)
ICU	0	2 (1.8)
Subspecialty clinic (outpatient)	0	10 (9.0)
Urgent care clinic	0	4 (3.8)

^a^The number of cases used in an academic year can vary slightly

^b^Federally qualified health center or safety net clinic

### Sustaining the Process

Our process allows incorporation of the optimal core attributes of the CBL case catalog listed above, making it more intentional and comprehensive for students’ development. This collaborative process initially required significant investments of time and energy on the part of many faculty. It also required a strong commitment on the part of the COT to prevent excessive lengthening of the cases and to maintain internal consistency while making necessary revisions. However, after this “overhaul” period, rates of case replacement have lessened, and the number of faculty needed to efficiently do the work of the team has decreased (from a maximum of 12 to 9 faculty). We also plan to deeply review only a portion of the cases each year on a rotational basis, which will reduce the overall workload while maintaining a high-quality case catalog. In the 4 years that our COT has been operating, 20 different faculty members have been part of the team with two different chairs, and many more faculty have participated in editing teams, provided comments, and/or provided expert content review.

In order to make the process sustainable, we plan to rotate the chair every 3 to 5 years, and we generally rotate only 1–3 members on and off the COT committee yearly so there is always a core of experienced COT faculty available to assist and train new members. At our institution, the position of COT chair requires 0.25–0.33 FTE and the lead editors (of which we have 7–8) require 0.1 FTE each. This investment is strongly supported by the faculty, as well as the administration, given the prominence of the cases in our pre-clerkship curriculum. The chair and members receive recognition for their service on the COT, as well as their contributions to curriculum development, in their annual reviews.

Students continue to respond favorably to the CBL portion of the curriculum. Further studies are underway to capture specific feedback on case changes from faculty and 3^rd^- and 4^th^-year medical students. These qualitative studies will allow us to further refine our process as needed. Anecdotally, many faculty have expressed a greater appreciation for the overall progression of the pre-clerkship curriculum. Additionally, faculty have more input into CBL which may lead to greater engagement and job satisfaction.

## Conclusions

The institutional process described here produces patient-centered and mission-centered cases that weave together many threads of our pre-clerkship curriculum, and which are intentionally targeted towards the progressive development of our student learners. These case catalog attributes and method of continuous quality improvement could be applied in a variety of other circumstances, including:

- Creating robust and diverse case catalogs at new medical schools or existing institutions that are transitioning to or expanding case-based learning;
- Systematically integrating clinical and/or foundational science facets in existing case catalogs;
- Intentionally making existing case catalogs more diverse and inclusive; and
- Revising other types of curricula, including simulation, flipped-classroom exercises, team-based learning, clerkships, graduate medical education, and other health professional curricula.
